# Spleen Volume Variation in Patients with Locally Advanced Non-Small Cell Lung Cancer Receiving Platinum-Based Chemo-Radiotherapy

**DOI:** 10.1371/journal.pone.0142608

**Published:** 2015-11-24

**Authors:** Shu Wen Wen, Sarah J. Everitt, Justin Bedő, Marine Chabrot, David L. Ball, Benjamin Solomon, Michael MacManus, Rodney J. Hicks, Andreas Möller, Antoine Leimgruber

**Affiliations:** 1 Tumour Microenvironment Laboratory, QIMR Berghofer Medical Research Institute, Herston, QLD, Australia; 2 Department of Radiation Oncology, Division of Radiation Oncology and Cancer Imaging, Peter MacCallum Cancer Centre, East Melbourne, VIC, Australia; 3 The Sir Peter MacCallum Department of Oncology, The University of Melbourne, Parkville, VIC, Australia; 4 Department of Medical Imaging and Radiation Sciences, Monash University, Clayton, VIC, Australia; 5 IBM Research—Australia, Carlton, VIC, Australia; 6 Department of Computing and Information Systems, The University of Melbourne, Parkville, VIC, Australia; 7 Centre for Molecular Imaging, Division of Radiation Oncology and Cancer Imaging, Peter MacCallum Cancer Centre, East Melbourne, VIC, Australia; 8 Medical Oncology, Peter MacCallum Cancer Centre, East Melbourne, VIC, Australia; 9 School of Medicine, University of Queensland, Brisbane, QLD, Australia; 10 Department of Medical Imaging, Centre Hospitalier Universitaire Vaudois and University of Lausanne, Lausanne, Switzerland; INRS, CANADA

## Abstract

There is renewed interest in the immune regulatory role of the spleen in oncology. To date, very few studies have examined macroscopic variations of splenic volume in the setting of cancer, prior to or during therapy, especially in humans. Changes in splenic volume may be associated with changes in splenic function. The purpose of this study was to investigate variations in spleen volume in NSCLC patients during chemo-radiotherapy. Sixty patients with stage I-IIIB NSCLC underwent radiotherapy (60Gy/30 fractions) for six weeks with concomitant carboplatin/paclitaxel (Ca/P; n = 32) or cisplatin/etoposide (Ci/E; n = 28). A baseline PET/CT scan was performed within 2 weeks prior to treatment and during Weeks 2 and 4 of chemo-radiotherapy. Spleen volume was measured by contouring all CT slices. Significant macroscopic changes in splenic volume occurred early after the commencement of treatment. A significant decrease in spleen volume was observed for 66% of Ca/P and 79% of Ci/E patients between baseline and Week 2. Spleen volume was decreased by 14.2% for Ca/P (p<0.001) and 19.3% for Ci/E (p<0.001) patients. By Week 4, spleen volume was still significantly decreased for Ca/P patients compared to baseline, while for Ci/E patients, spleen volume returned to above baseline levels. This is the first report demonstrating macroscopic changes in the spleen in NSCLC patients undergoing radical chemo-radiotherapy that can be visualized by non-invasive imaging.

## Introduction

The spleen is involved as a predominant feature of many pathological conditions and there is renewed interest in its function in oncology [1]. The size and shape of the spleen is assessed most often and reliably by computed tomography (CT) and ultrasonography [2]. Clinically, assessment of splenic volume is important in the diagnosis, treatment and prognosis of diseases such as lymphoma, leukemia and other hematologic neoplasms [3,4]. Malignant splenic infiltration often leads to splenomegaly (splenic enlargement). More recently, there has been increasing interest in the impact of chemotherapy on modulating splenic activity, given the significant adverse effects of chemotherapy on hematopoiesis and immunocompetence. Indeed, several conventional chemotherapeutics have already been shown to alter the splenic niche [5,6]. For example, low dose administration of several chemotherapeutic agents such as gemcitabine, fludarabine and 5-fluorouracil in tumor-bearing hosts can prevent the accumulation of myeloid derived suppressor cells (MDSCs) by restoring CD8+ T cells in the splenic niche [5]. In patients without gross tumor infiltration of the spleen, (which is uncommon in epithelial cancers) changes in splenic volume during treatment may be correlated with alterations in the number and function of immune effector cells in the spleen.

For patients with unresectable, locally-advanced non-small cell lung carcinoma (NSCLC), radiotherapy given with concurrent chemotherapy is the standard of care [7]. At our center, the chemotherapy component of the chemoradiation protocol commonly consists of a standard combination of cisplatin and etoposide (Ci/E), or carboplatin and paclitaxel (Ca/P) in less fit patients. We recently reported that positron emission tomography with computed tomography (PET/CT) imaging using the radiotracers ^18^F-3′-deoxy-3′-fluoro-l-thymidine (FLT) and ^18^F-fluoro-2′-deoxy-d-glucose (FDG) can monitor and quantify organ-specific processes, including metabolic and proliferative activity in the spleen during and after chemo-radiotherapy in patients with NSCLC [8]. To date, no studies that we are aware of have documented the effect of chemotherapy on spleen volume as a surrogate of splenic activity in patients with NSCLC. Therefore, the purpose of this study was to measure and quantify temporal changes in spleen volume *in vivo* on a prospective trial in NSCLC patients receiving chemo-radiotherapy. In this ongoing trial (Registry#ACTRN12611001283965), patients with stage I-IIIB NSCLC are treated with either one of two chemotherapy regimens: cisplatin/etoposide or carboplatin/paclitaxel [8]. Serial CT imaging, available at PET/CT examination time points (baseline and in-treatment) was used as an accurate non-invasive means to estimate spleen volume.

## Materials and Methods

### Patients and treatment

Patients with stage I-IIIB NSCLC considered by our multi-disciplinary tumor board to be suitable candidates for radical chemo-radiotherapy were recruited between March 2009 and June 2013 for this ongoing trial (Registry#ACTRN12611001283965). Patients with prior thoracic radiotherapy or complete macroscopic tumor excision were excluded. This trial was approved by our institutional ethics committee (Peter MacCallum Cancer Centre, Melbourne, Australia), and written, informed consent was provided by patients. Patients were treated with conformal radiotherapy (60Gy/30 fractions) for six weeks with concomitant carboplatin/paclitaxel (Ca/P) or cisplatin/etoposide (Ci/E). The Ca/P regime was administered weekly and consisted of carboplatin (dosage as determined from renal function with a target area-under-curve of 2 mg/ml min) and paclitaxel (45 mg/m^2^). The Ci/E regime consisted of cisplatin (50 mg/m^2^) on days 1&8 (cycle 1; week 1) and 29&36 (cycle 2; week 5), and etoposide (50 mg/m^2^) daily during cycle 1 (day 1–5) and cycle 2 (day 29–33). Additionally, dexamethasone was administered intravenously at 20 mg for Ca/P, and 12 mg for Ci/E.

### Imaging and spleen CT measurement

Free-breathing PET/CT imaging was performed at 3 time points: baseline, week 2 (day 9 for FLT and day 10 for FDG) and week 4 (day 23 for FLT and day 24 for FDG). Scans were acquired on a GE/STE or Biograph PET/CT modality (GE Medical Systems Milwaukee, WI, USA, Siemens Medical Solutions, Erlangen, Germany). Spleen CT measurements where performed on the CT component of the FLT scans (helical 140kV slice thickness 2.5mm). Baseline FLT scans were performed from vertex to thighs. To reduce radiation exposure, week 2 and week 4 scans were limited from thoracic inlet to mid-abdomen. CT spleen volume and size measurements were performed on an Advantage Windows4.0 workstation (GE, Milwaukee, WI, USA). Spleen volume was obtained by contouring manually all axial slides. Maximal splenic width (W) and diameter (T2) where measured manually as previously described [9–11].

### Statistical Analysis

The time series data was analyzed using mixed-effect modeling [12]. Patient and group variance was modeled as random effects, and weeks 2 and 4 were treated as fixed effects factors over the baseline at week 0. P-values were calculated for the mixed-effect model using Satterthwaite's approximations [13]. All calculations were done using the lm4r and lmerTest packages in R. P-values were adjusted for multiple testing using Holm correction [14] and a P-value less than 0.05 was considered statistically significant.

## Results

### Patient cohort

The demographic and clinical characteristics of patients in this study are depicted in Table 1. Sixty-seven eligible patients with stage I-IIIB NSCLC were recruited between March 2009 and June 2013 for the study. Of those patients, seven were excluded because they withdrew consent prior to baseline scan or were considered unsuitable after multidisciplinary review of disease burden. A total of 60 patients were retained: 37% (22/60) female and 63% (38/60) male. The median age of female and male patients was 63 and 69 respectively. Tumors present in patients were grouped into five histology categories: adenocarcinoma in 45% (27/60), squamous cell carcinoma in 35% (21/60), large cell carcinoma in 10% (6/60), non-specified in 7% (4/60) and neuroendocrine component in 3% (2/60). The majority of patients presented with AJCC stage III disease (75%, 45/60) and radiotherapy included the primary tumor and involved mediastinal and hilar lymph nodes. Bone marrow in the thoracic spine, ribs and scapulae received direct or scattered radiation from radiotherapy. Of the 60 patients, 47% (28/60) received the cisplatin/etoposide (Ci/E) regimen and 53% (32/60) the carboplatin/paclitaxel (Ca/P) regime.

**Table 1:** Background characteristics of the patient cohort with stage I-IIIB NSCLC included in this study and candidates for radical chemo-radiotherapy (carboplatin/paclitaxel or cisplatin/etoposide). Quantitative data are presented as means ± S.E.M.

	Carboplatin/Paclitaxel (Ca/P) (n = 32)	Cisplatin/Etoposide (Ci/E) (n = 28)
Gender (male/female)	23/9	15/13
Age	70.4±1.8	62.3±1.8
Histology	AdenoCA (n = 11)	AdenoCA (n = 16)
	Large Cell (n = 3)	Large Cell (n = 3)
	SCC (n = 15)	SCC (n = 6)
	Neuroendocrine (n = 1)	Neuroendocrine (n = 1)
	Non-specified (n = 2)	Non-specified (n = 2)

### Macroscopic variations of spleen volume during chemo-radiotherapy

**Fig 1:**
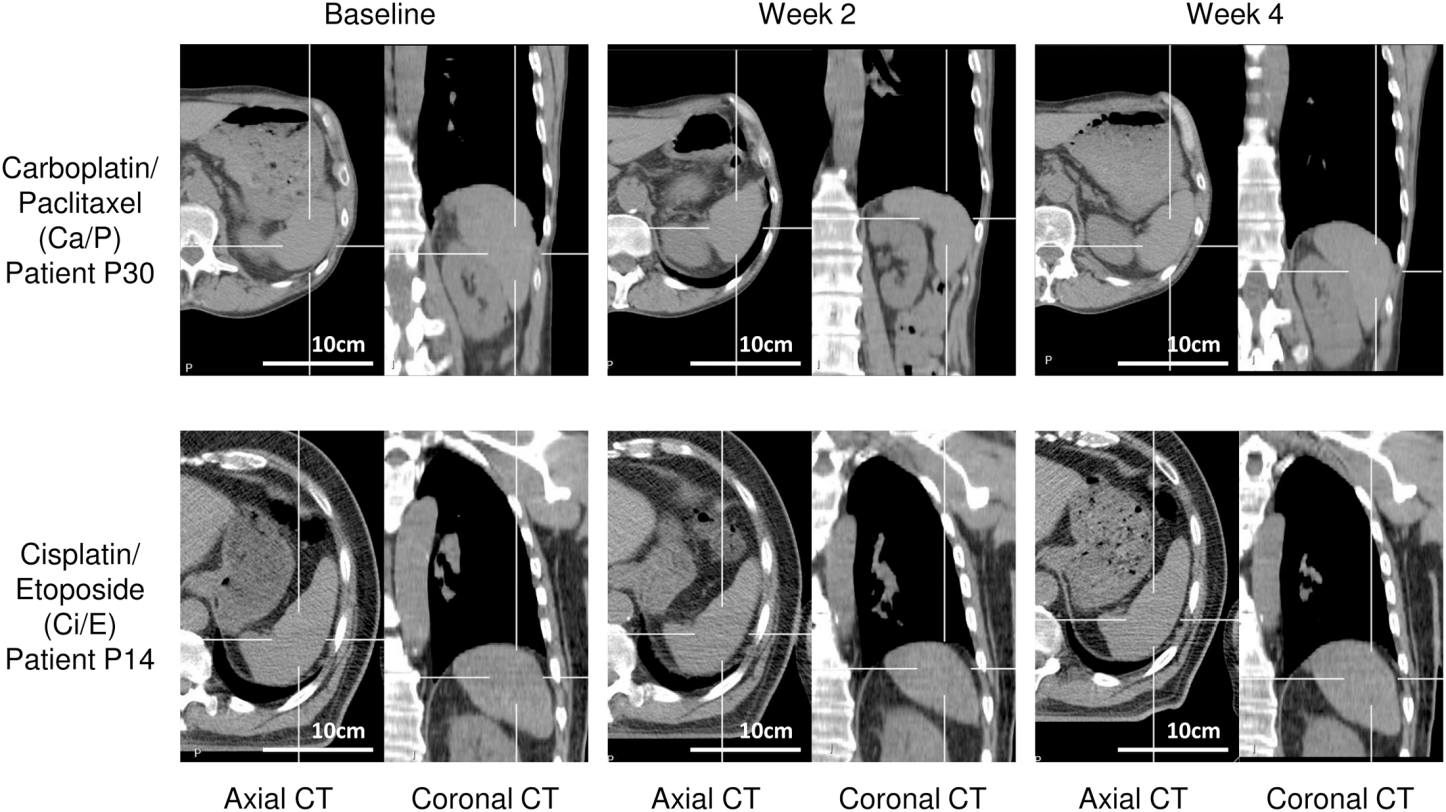
Representative computed tomography (CT) images used to calculate spleen volume in patients treated with carboplatin/paclitaxel (Ca/P) or cisplatin/etoposide (Ci/E). CT imaging was performed at baseline, Week 2 and Week 4 of chemo-radiotherapy.

Assessment of patient spleen size at baseline, week 2 and week 4 of chemo-radiotherapy [(Ca/P) or (Ci/E)] using CT demonstrated significant visible volume fluctuations during chemotherapy (Fig 1).

**Fig 2:**
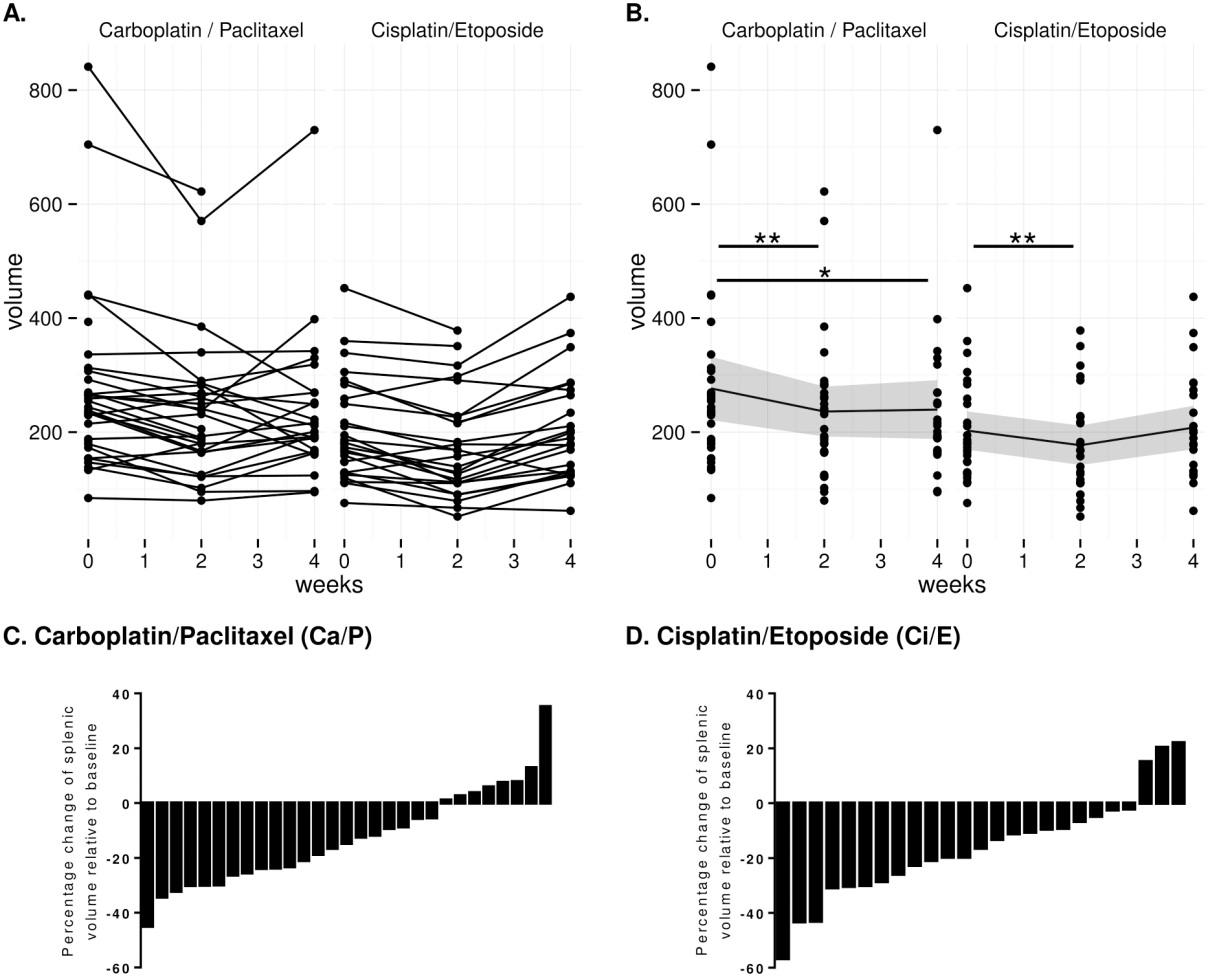
Changes in spleen volume at baseline, Week 2 and Week 4 of chemo-radiotherapy with carboplatin/paclitaxel (Ca/P) or cisplatin/etoposide (Ci/E). (A) Changes in the spleen volume of each individual patient. (B) Mean change of spleen volume and 95% confidence interval of the mean (dark grey ribbon). In Ca/P and Ci/E treatment regimes, spleen volume was significantly decreased at Week 2. (C and D) Percentage change of spleen volume at Week 2 of chemo-radiotherapy relative to baseline for individual patients. *p<0.05, ** p<0.001.

The mean spleen volume before chemotherapy (baseline) was 276.1±21.2 mm^3^. The Ci/E patients had a smaller spleen size at baseline (-71.9±31.1 mm^3^, p<0.05). Despite this initial difference in spleen volume, 66% (21/32) of Ca/P and 79% (22/28) of Ci/E patients (Fig 2A–2D) showed decreased spleen volume after treatment at week 2 compared to baseline. Spleen volume decreased by -39.2±10.0 mm^3^ (p<0.001) at week 2 for both Ca/P (14.2% decrease) and Ci/E patients (19.3% decrease) (Fig 2B). There was no significant difference between treatments at week 2 (p = 0.53).

In week 4 there was still a significant decrease in spleen volume of -26.5±10.9 mm^3^ (p<0.05) compared to baseline (276.1±21.2 mm^3^) for Ca/P patients (9.6% decrease) (Fig 2B). A significant increase in spleen volume of 43.4±15.7 mm^3^ (p<0.03) was observed in Ci/E compared to Ca/P patients at week 4. This resulted in a non-significant estimated increase in volume of 16.8±19.1mm^3^ over baseline for Ci/E patients (Fig 2B).

**Fig 3:**
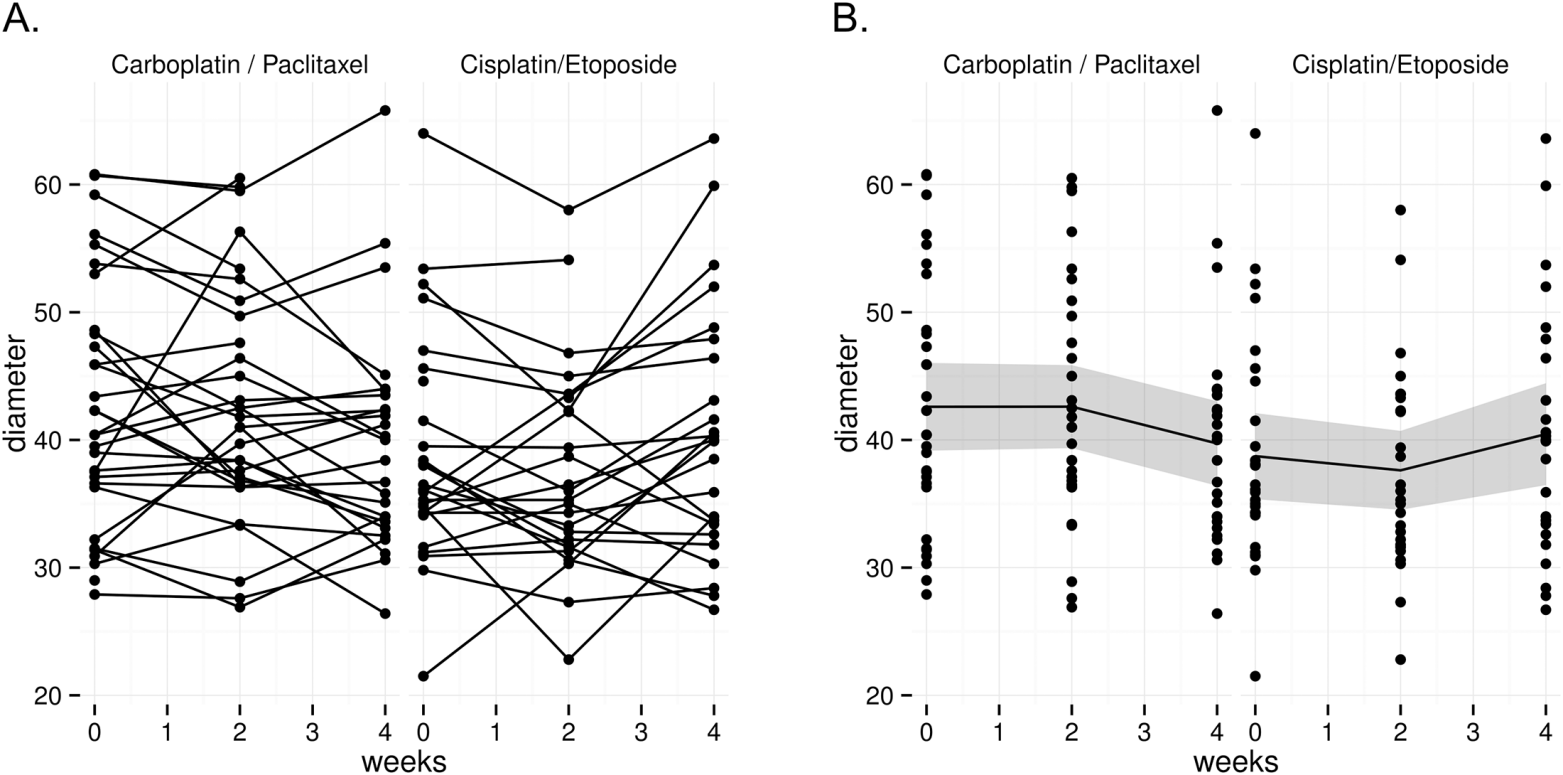
Changes in spleen diameter at baseline, Week 2 and Week 4 of chemo-radiotherapy with carboplatin/paclitaxel (Ca/P) or (B) cisplatin/etoposide (Ci/E). (A) Changes in the spleen diameter of each individual patient. (B) Mean change of spleen diameter and 95% confidence interval of the mean (dark grey ribbon).

Unlike the variations in spleen volume, spleen diameter did not show significant changes at week 2 and week 4 compared to baseline for both Ci/P and Ca/E groups (Fig 3).

## Discussion

Chemo-radiotherapy is currently the mainstay of treatment for patients with potentially curable but unresectable locally advanced NSCLC, with a median overall survival of approximately 24 months reported in recent series [15–17]. Monitoring the impact of chemotherapy on the spleen—an organ known to regulate hematopoiesis and immune responses [1]—is of interest in the context of the increasing appreciation of immunotherapy in NSCLC and other epithelial malignancies. To date, few studies have evaluated variations in spleen size as a potential indicator of changes in immune function during chemotherapy and its clinical significance [18]. This analysis, derived from an ongoing prospective clinical study, showed, for the first time that significant macroscopic changes occur in the human spleen early after the commencement of platinum-based chemoradiation in patients with NSCLC.

The change in spleen volume was observed to be a dynamic process. Patients treated with either Ca/P or Ci/E induced an initial early decrease in spleen volume, which subsequently recovered to baseline levels for Ci/E treatment. Differences in treatment regimes between Ca/P and Ci/E patients may, in part, determine if spleen volumes return to baseline levels by week 4: Ca/P patients received uninterrupted weekly chemotherapy while Ci/E patients had a 2-weeks pause in chemotherapy with ongoing radiotherapy between the week 2 and week 4 scans. Our results also suggest that spleen size is reduced for longer in Ca/P patients. All patients were treated according to standard radiotherapy dose and fractionation prescriptions, indicating that variation in spleen volume for Ci/E and Ca/P groups were not dependent on radiotherapy effects. Using the same patient cohort as described in the present study, we have demonstrated previously the capability of FLT-PET/CT imaging to indirectly monitor proliferative activity in the spleen during chemotherapy [8]. Recovery of spleen volume and splenic FLT uptake was accompanied by increased circulating monocytes [8]. Recent studies in murine models of lung carcinoma showed that the spleen is a reservoir for the expansion of splenic myeloid progenitors and can significantly contribute to the host response to impair tumor progression [19]. Similarly, in a mouse model of colorectal cancer, splenectomy was shown to increase liver metastasis suggesting the importance of the spleen in initiating anti-tumor immune responses [20]. It would therefore be important to understand whether chemotherapeutic agents, individually or in combination, can enhance hematopoietic and immune responses in the spleen to favor host survival. Our results demonstrate that aspects of splenic function can be visualized indirectly and evaluated in cancer patients by non-invasive radiological and molecular imaging technologies. Furthermore, we have confirmed that changes in spleen volume occur in the setting of myelotoxic cytotoxic chemoradiation regimens. This study has the potential to link studies of tumor related immunity in small animals to the bedside.

Accurate evaluation of spleen size has clinical importance as an indicator of disease activity in a number of disorders [21]. To date, splenomegaly (enlargement of spleen) has received most attention in inflammatory, metabolic and malignant diseases that directly involve the immune system, liver and hematopoietic system [2–4]. Results from the present study demonstrate that diminution in spleen size can occur in patients with epithelial cancer during therapy and together with future functional investigations, this may improve our understanding of dynamic changes in the immune environment of tumors during treatment. Several techniques have been used to determine spleen size, including nuclear scintigraphy, sonography, magnetic resonance imaging and CT. CT is considered to offer the most rapid and reliable non-invasive method of estimating volume of the spleen with the highest sensitivity and specificity [11,22–24]. This is because CT allows clinicians to precisely define organ contours and can easily scan organs in their entirety. The current study employed a method previously described to accurately estimate spleen volume using linear measurements (length, width and thickness) obtained from axial CT images [9–11]. While spleen length and multidimensional indexes have been previously shown to correlate well with spleen CT volume in the setting of splenomegaly [11], this was not the case in the current study. We found that unlike spleen volume, spleen diameter measurements alone were insufficient to demonstrate spleen macroscopic changes in NSCLC patients during chemo-radiotherapy. Therefore, our results indicate that in a clinical setting both spleen volume and spleen diameter should be taken into consideration when monitoring spleen size during pathology. Although we have demonstrated significant changes in spleen volume during treatment we have no information on the mechanism of these changes. This finding paves the way for future research on this complex organ that is composed of many cellular components and phenotypes that are sensitive to chemotherapy.

In conclusion, we demonstrate for the first time dynamic temporal changes in human spleen volume in patients receiving chemoradiation for NSCLC. Future correlative studies would be of interest to assess if these changes in spleen volume are associated with changes in other immune parameters, or if it can be used as a predictor of patient outcome or the efficacy of chemo-radiotherapy.

## References

[1] Bronte V, Pittet MJ (2013) The spleen in local and systemic regulation of immunity. Immunity 39: 806–818. 10.1016/j.immuni.2013.10.010 24238338 PMC3912742

[2] Pozo AL, Godfrey EM, Bowles KM (2009) Splenomegaly: investigation, diagnosis and management. Blood Rev 23: 105–111. 10.1016/j.blre.2008.10.001 19062140

[3] Koga T, Morikawa Y (1975) Ultrasonographic determination of the splenic size and its clinical usefulness in various liver diseases. Radiology 115: 157–161. 1090976 10.1148/115.1.157

[4] Griffith R, Jnney C (1990) Hematopoietic system: bone marroe and blood, spleen and lymph nodes Anderson's pathology. St Louis: Mosby.

[5] Ugel S, Peranzoni E, Desantis G, Chioda M, Walter S, Weinschenk T, et al (2012) Immune tolerance to tumor antigens occurs in a specialized environment of the spleen. Cell Rep 2: 628–639. 10.1016/j.celrep.2012.08.006 22959433

[6] Germano G, Frapolli R, Belgiovine C, Anselmo A, Pesce S, Liguori M, et al (2013) Role of macrophage targeting in the antitumor activity of trabectedin. Cancer Cell 23: 249–262. 10.1016/j.ccr.2013.01.008 23410977

[7] Price A (2012) Emerging developments of chemoradiotherapy in stage III NSCLC. Nat Rev Clin Oncol 9: 591–598. 10.1038/nrclinonc.2012.135 22926022

[8] Leimgruber A, Moller A, Everitt SJ, Chabrot M, Ball DL, Solomo B, et al (2014) Effect of Platinum-Based Chemoradiotherapy on Cellular Proliferation in Bone Marrow and Spleen, Estimated by 18F-FLT PET/CT in Patients with Locally Advanced Non-Small Cell Lung Cancer. J Nucl Med 55: 1075–1080. 10.2967/jnumed.113.136127 24868108

[9] Schlesinger AE, Hildebolt CF, Siegel MJ, Pilgrim TK (1994) Splenic volume in children: simplified estimation at CT. Radiology 193: 578–580. 7972783 10.1148/radiology.193.2.7972783

[10] Cools L, Osteaux M, Divano L, Jeanmart L (1983) Prediction of splenic volume by a simple CT measurement: a statistical study. J Comput Assist Tomogr 7: 426–430. 6841704 10.1097/00004728-198306000-00007

[11] Bezerra AS, D'Ippolito G, Faintuch S, Szejnfeld J, Ahmed M (2005) Determination of splenomegaly by CT: is there a place for a single measurement? AJR Am J Roentgenol 184: 1510–1513. 15855107 10.2214/ajr.184.5.01841510

[12] Gałecki A, Burzykowski T (2013) Linear Mixed-Effects Models Using R: A Step-by-Step approach Springer Texts in Statistics: Springer pp. 67.

[13] Satterthwaite FE (1946) An Approximate Distribution of Estimates of Variance Components. Biometrics Bulletin 2: 110–114. 20287815

[14] Holm S (1979) A simple sequentially rejective multiple test procedure. Scandinavian Journal of Statistics 6: 65–70.

[15] Stinchcombe TE, Lee CB, Moore DT, Rivera MP, Halle J, Limentani S, et al (2008) Long-term follow-up of a phase I/II trial of dose escalating three-dimensional conformal thoracic radiation therapy with induction and concurrent carboplatin and paclitaxel in unresectable stage IIIA/B non-small cell lung cancer. J Thorac Oncol 3: 1279–1285. 18978563 10.1097/JTO.0b013e31818b1971

[16] Socinski MA, Blackstock AW, Bogart JA, Wang X, Munley M, Roseman J, et al (2008) Randomized phase II trial of induction chemotherapy followed by concurrent chemotherapy and dose-escalated thoracic conformal radiotherapy (74 Gy) in stage III non-small-cell lung cancer: CALGB 30105. J Clin Oncol 26: 2457–2463. 10.1200/JCO.2007.14.7371 18487565

[17] Schild SE, McGinnis WL, Graham D, Hillman S, Fitch TR, Northfelt D, et al (2006) Results of a Phase I trial of concurrent chemotherapy and escalating doses of radiation for unresectable non-small-cell lung cancer. Int J Radiat Oncol Biol Phys 65: 1106–1111. 16730134 10.1016/j.ijrobp.2006.02.046

[18] Imai K, Emi Y, Iyama KI, Beppu T, Ogata Y, Kakeji Y, et al (2014) Splenic volume may be a useful indicator of the protective effect of bevacizumab against oxaliplatin-induced hepatic sinusoidal obstruction syndrome. Eur J Surg Oncol 40: 559–566. 10.1016/j.ejso.2013.12.009 24388740

[19] Cortez-Retamozo V, Etzrodt M, Newton A, Rauch PJ, Chudnovskiy A, Berger C, et al (2012) Origins of tumor-associated macrophages and neutrophils. Proc Natl Acad Sci U S A 109: 2491–2496. 10.1073/pnas.1113744109 22308361 PMC3289379

[20] Higashijima J, Shimada M, Chikakiyo M, Miyatani T, Yoshikawa K, Nishioka M, et al (2009) Effect of splenectomy on antitumor immune system in mice. Anticancer Res 29: 385–393. 19331177

[21] Saboo SS, Krajewski KM, O'Regan KN, Giardino A, Brown JR, Ramaiya N, et al (2012) Spleen in haematological malignancies: spectrum of imaging findings. Br J Radiol 85: 81–92. 10.1259/bjr/31542964 22096219 PMC3473934

[22] Yetter EM, Acosta KB, Olson MC, Blundell K (2003) Estimating splenic volume: sonographic measurements correlated with helical CT determination. AJR Am J Roentgenol 181: 1615–1620. 14627584 10.2214/ajr.181.6.1811615

[23] Lamb PM, Lund A, Kanagasabay RR, Martin A, Webb JA, Reznek RH (2002) Spleen size: how well do linear ultrasound measurements correlate with three-dimensional CT volume assessments? Br J Radiol 75: 573–577. 12145129 10.1259/bjr.75.895.750573

[24] Breiman RS, Beck JW, Korobkin M, Glenny R, Akwari OE, Heaston DK, et al (1982) Volume determinations using computed tomography. AJR Am J Roentgenol 138: 329–333. 6976739 10.2214/ajr.138.2.329

